# Prevalence and Predictors of Peer Physical Violence Among Adolescents in a Sub-National Region of Nigeria

**DOI:** 10.3389/ijph.2025.1608128

**Published:** 2025-07-17

**Authors:** Grace I. Nwankwo, Ogonna N. O. Nwankwo, Onyinye U. Anyanwu, Chinonyelum T. Ezeonu, Chigozie I. Uzomba, Michael A. Akpoke, Anthony N. Ikefuna

**Affiliations:** ^1^ Department of Paediatrics, University of Calabar Teaching Hospital, Calabar, Nigeria; ^2^ Swiss Centre for International Health, Swiss Tropical and Public Health Institute (Swiss TPH), Basel, Switzerland; ^3^ University of Basel, Basel, Switzerland; ^4^ Department of Community Medicine, University of Calabar, Calabar, Nigeria; ^5^ Department of Public Health and Community Medicine, University of Calabar Teaching Hospital, Calabar, Nigeria; ^6^ Department of Paediatrics, Alex Ekwueme Federal University Teaching Hospital Abakaliki, Abakaliki, Nigeria; ^7^ Institute of Child Health, Alex Ekwueme Federal University Teaching Hospital, Abakaliki, Nigeria; ^8^ Department of Paediatrics, Faculty of Medicine, University of Calabar, Cross River, Nigeria; ^9^ Department of Paediatrics, University of Sierra Leone Teaching Hospital, Freetown, Sierra Leone; ^10^ Department of Paediatrics and Child Health, University of Nigeria, Enugu, Nigeria; ^11^ Department of Paediatrics, University of Nigeria Teaching Hospital, Enugu, Nigeria

**Keywords:** adolescent, physical fight, Nigeria, violence, bullying

## Abstract

**Objective:**

Peer physical violence (PPV) has been shown to be an early marker for development of other health-risk behaviours. This study assessed the prevalence and risk factors of PPV among in-school adolescents in a state in South-east, Nigeria.

**Methods:**

This was a cross sectional study conducted among 1,296 in-school adolescents using the Global School-based students Health Survey questionnaire. Data on socio-demographic characteristics and factors associated with PPV were obtained and p-value <0.05 was considered significant.

**Result:**

The overall mean age (S.D) of participants was 15.0 ± 2.0 years and the prevalence of PPV was 43.1%. In multivariate logistic regression, predictors of PPV were gambling (AOR: 1.56; 95%CI:1.13–2.16; p = 0.007), cigarette smoking (AOR: 1.85; 95%CI:1.01–3.40; p = 0.047), serious injury in the past 1 year (AOR: 2.29; 95%CI:1.78–2.95; p < 0.001) and bully victims (AOR: 1.70; 95% CI:1.28–2.25; p < 0.001). Older adolescent age (AOR:0.37; 95%CI:0.25–0.53; p < 0.001] and being religious (AOR: 0.70; 95%CI: 0.53–0.92; p = 0.011) were protective.

**Conclusion:**

There is high prevalence of PPV in the study population. The risk factors were young adolescence age, bullying, gambling, cigarette smoking, having had a serious injury and not being religious. Stricter regulations on gambling through legislation, especially as it concerns age, and adoption of school policies against bullying and cigarette smoking are recommended.

## Introduction

Adolescents are young people aged 10–19 years [1], and make up one-sixth (16.7%) of the world population, numbering 1.2 billion people [2]. This proportion is higher for developing countries such as Nigeria, where up to 21% of the population are adolescents [2, 3]. The period of adolescence is often a stressful period [4]; where many risky health-related behaviours can begin, as adolescents start to experience more independence and increased peer pressure [5, 6]. Peer physical violence differs from bullying and occurs when ‘two people of about the same strength and power choose to fight each other’ [7]. Globally, the prevalence of peer physical violence amongst adolescents varies, ranging from 15.9% in Myanmar, Asia to 57.7% in Djibouti, Northeast Africa [8–12].

Adolescents who engage in peer physical violence are more likely to have serious injury requiring medical treatment [13, 14]. A study carried out in 35 high-income countries among adolescents, consistently showed that peer physical violence was significantly associated with elevated risks of multiple, medically treated and hospitalized injuries [13]. This leads to higher healthcare cost for households and governments. Estimates has been made that more than $3 billion is spent on hospital treatment for non-fatal injuries resulting from fighting among young people in the United States of America [15]. Globally, there have been calls for concerted public health efforts including research, to reduce violence among adolescents, through identifying individual and social conditions that predispose them to violent behaviour [16, 17]. However, most studies addressing this subject matter have largely been from high-income countries, with fewer studies emanating from low and middle-income countries like Nigeria. The available data from the high-income countries may not provide the optimal framework and contextual evidence to assist in proffering solutions to this issue, hence the need for this study. Peer physical violence has also been shown to be an early marker for development of other health-risk behaviours in adolescents [16, 18]. Providing data on these unhealthy behaviours would be a step towards curbing them too. This study aimed to determine the prevalence, and factors associated with peer physical violence among in-school adolescents in a state in the South-eastern region of Nigeria.

## Methods

This was carried out as a descriptive cross-sectional survey among in-school adolescents in Ebonyi State.

### Setting

Ebonyi State is in the south-east region of Nigeria and is primarily an agricultural region, with an estimated population of 3,140,944 [19]. According to the Ebonyi State Ministry of Education, there are 299 registered secondary schools with a total school population of 367,001 and also higher number of public schools.

### Participants

In-school adolescents aged 10–19 years were recruited using a multi-stage sampling method. Out of 13 local government areas (LGAs) in Ebonyi state, four were selected using simple random sampling by balloting; with two being rural and two being urban. In each of the selected LGA, three secondary schools were selected by simple random sampling from the list of secondary schools. One private and two public schools were selected from each L.G.A using a proportion of 1:2. Equal number of students (108) were allocated to each school from the sample size. From the earlier selected schools, a class from each level of study was selected randomly. However, for schools with only one class per level, the class was automatically selected. Subsequently, participants were proportionately allocated to every level of study in each school. All eligible students in the class were consecutively recruited into the study until the allocated sample size was met. For each level of study, all recruited participants were seated well-spaced from one another in a large classroom and the questionnaire self-administered with no interference from the investigator. These sessions lasted approximately 30 min each.

### Sample Size Determination

Sample size was calculated using Cochran formula for sample size for single proportion, considering 95% significance level, 3% margin of error(d) and an estimated proportion of 35% of PPV drawn from a previous study [20], while accounting for an expected 20% for non-response. This gave an estimated sample size of 1,217. Eventually, a total of 1,296 adolescents aged 10–19 years participated in the study; with a mean age of 15.0 ± 2.0 years and the proportion of males recruited 42%.

### Data Collection

Data was collected between November 2020 and January 2021, using a pretested self-administered WHO/CDC Global school-based Health Survey (GSHS) questionnaire as adapted. The adaptation occurred by modifying a few questions from the original GSHS questionnaire to fit the cultural context of the study area. For example, in the question on drug/substance use, the term “marijuana” in the GSHS questionnaire was modified to a different term (“Igbo”) which is more popularly used within the study area. Furthermore, the language for some of the questions were rephrased for better understanding following a pre-test conducted.

### Ethical Consideration

Ethical approval was obtained from the Research and Ethics Committee of the Federal Teaching Hospital, Abakaliki, Ebonyi State (FETHA/REC/VOL2/2019/165). Permission was obtained from the school authorities. Written informed consent and assent were obtained from the parents and participants respectively.

### Dependent and Independent Variables

The dependent variable – peer physical violence, was assessed using the question, “During the past 12 months, how many times were you in a physical fight?” Participants who had not been in a physical fight were coded as “0 = No” while those involved in 1 or more fights were coded as “1 = Yes.”

Independent variables including family socioeconomic status (as derived based on methods proposed by Olusanya and colleagues) [21], drug use, smoking, gambling, religiosity, bullying, suicide attempt, sexual exposure, weapon carrying, serious injury and alcohol use as described in Supplementary Appendix S1, were assessed.

### Statistical Analysis

All quantitative analyses were done using STATA software (Version 18; College Station, TX:Statacorp LLC). Descriptive analysis was done using mean and the standard deviation for continuous variables, while proportions and frequency were used to describe the categorical variables as when appropriate. Tables were used to display some of the data. Univariate and multivariate binary logistic regression models were fitted to determine factors associated with peer physical violence (PPV). The relationship between each of the independent variables and PPV was analysed in the unadjusted regression analysis at 5% significance level. The independent variables that were not significant in the unadjusted analysis were excluded for analysis in the adjusted model while the variables that were significantly associated (confidence intervals excluding the null value of one) with PPV in the unadjusted analysis were included in the final adjusted regression model at 5% significance level.

## Results

A total of 1,296 adolescents participated in the study, out of which 991 (76.5%) were older than 13 years. The mean age of study participants was 15.0 ± 2.0 years. Majority (57.9%) of the participants were females. Overall, 559 (43.1%) adolescents were involved in peer physical violence (Tables 1, 2).

**TABLE 1:** Prevalence of Peer Physical Violence and Distribution of Socio-demographic characteristics of adolescents attending secondary schools. N = 1,296 (Prevalence and Predictors of Peer Physical Violence among Adolescents in a Sub-national Region of Nigeria, Abakaliki, Nigeria; January 2021).

Variable	Frequency (N = 1,296)	Percentage
Age/years		
10–13	305	23.5
14–16	692	53.4
17–19	299	23.1
Sex		
Male	545	42.1
Female	751	57.9
Family socioeconomic class		
Upper	548	42.3
Middle	233	18.0
Lower	515	39.7
School Type Public	864	66.7
Private	432	33.3
Study site Rural	648	50.0
Urban	648	50.0
Physical Fighting		
No	737	56.9
Yes	559	43.1

**TABLE 2:** Univariate analysis of relationship between Socio-demographic characteristics and Peer Physical Violence among the study participants. N = 1,296 (Prevalence and Predictors of Peer Physical Violence among Adolescents in a Sub-national region of Nigeria, Abakaliki, Nigeria; January 2021).

Variable	Peer physical violence		Univariate regression Unadjusted OR (95% CI)	p-value
	Present (n = 559) n (%)	Absent (n = 737) n (%)		
Age in years				
10–13	167 (54.8)	138 (45.2)	1	
14–16	300 (43.4)	392 (56.6)	0.63 (0.48–0.83)	0.001^a^
17–19	92 (30.8)	207 (69.2)	0.38 (0.27–0.52)	<0.001^a^
Sex				
Male	247 (45.3)	298 (54.7)	1	
Female	312 (41.5)	439 (58.5)	0.86 (0.69–1.07)	0.175
Family’s socioeconomic status				
Upper	231 (42.2)	317 (57.8)	1	
Middle	107 (45.9)	126 (54.1)	1.17 (0.86–1.59)	0.970
Lower	221 (42.9)	294 (57.1)	1.03 (0.81–1.32)	0.250
School Type				
Public	371 (42.9)	493 (57.1)	1	
Private	188 (43.5)	244 (56.5)	1.00 (0.80–1.27)	0.978
Study site				
Urban Area	271 (41.8)	377 (58.2)	1	
Rural Area	288 (44.4)	360 (55.6)	1.11 (0.89–1.39)	0.340

^a^ Statistically significant.

### Predictors of Peer Physical Violence Among Adolescents in the Study

In the univariate binary logistic analysis, age, religiosity and all the measured health risk behaviours of the adolescents were predictive of engaging in PPV (Table 3).

**TABLE 3:** Univariate analysis of the relationship between adolescent health-risk behaviours, religiosity and Peer Physical Violence among adolescents. N = 1,296 (Prevalence and Predictors of Peer Physical Violence among Adolescents in a Sub-national Region of Nigeria, Abakaliki, Nigeria; January 2021).

Variable	Peer physical violence		Univariate Regression Crude OR (95% CI)	p-value
	Involved (n = 559) n (%)	Not involved (n = 737) n (%)		
Alcohol Intake				
Do not take alcohol	373 (38.9)	585 (61.1)	1	
Take alcohol	186 (55.0)	152 (45.0)	1.84 (1.44–2.36)	<0.001^a^
Drug/Substance Use				
Do not use drug/substances	428 (39.9)	644 (60.1)	1	
Use drugs/substances	131 (58.5)	93 (41.5)	2.15 (1.61–2.88)	<0.001^a^
Smoking				
Do not smoke	496 (40.9)	716 (59.1)	1	
Smoke	63 (75.0)	21 (25.0)	4.09 (2.50–6.66)	<0.001^a^
Passive smoking				
No	367 (50.1)	366 (49.9)	1	
Yes	214 (38.4)	344 (61.6)	1.61 (1.29–2.02)	<0.001^a^
Gambling				
Do not gamble	400 (38.4)	641 (61.6)	1	
Gamble	159 (62.4)	96 (37.6)	2.59 (1.96–3.43)	<0.001^a^
Sexual exposure				
Have not had sex	469 (41.6)	659 (58.4)	1	
Have had sex	84 (53.2)	74 (46.8)	1.60 (1.14–2.23)	0.006^a^
Weapon carrying				
Do not carry weapon	504 (41.5)	711 (58.5)	1	
Carry weapon	55 (67.9)	26 (32.1)	3.00 (1.87–4.81)	<0.001^a^
Serious injury				
Have not had serious injury	186 (30.1)	432 (69.9)	1	
Have had serious injury	373 (55.0)	305 (45.0)	2.86 (2.27–3.59)	<0.001^a^
Suicide attempt				
Not attempted suicide	485 (41.2)	693 (58.8)	1	
Attempted suicide	66 (65.73)	35 (34.7)	2.69 (1.76–4.12)	<0.001^a^
Bully victim				
Not a bully-victim	274 (33.5)	545 (66.5)	1	
Bully-victim	285 (59.7)	192 (40.3)	2.95 (2.34–3.73)	<0.001^a^
Bully perpetrator				
Not bullied someone	334 (35.1)	618 (64.9)	1	
Bullied someone	225 (65.4)	119 (34.6)	3.50 (2.70–4.53)	<0.001^a^
Religiosity				
Not religious	142 (53.6)	123 (46.4)	1	
Religious	417 (40.4)	614 (59.6)	0.64 (0.50–0.81)	<0.001^a^

^a^ Statistically significant.

In the multivariate binary logistic regression, adolescents aged 14–16 years (AOR: 0.63; 95% CI:0.46–0.85; p = 0.002) and 17–19 years (AOR:0.37; 95% CI: 0.25–0.53; p < 0.001) were significantly less likely to be involved in peer physical violence compared to the younger adolescents (10–13 years).

Adolescents involved in gambling (AOR: 1.56; 95% CI:1.13–2.16; p = 0.007), who smoke cigarettes (AOR: 1.85; 95% CI:1.01–3.40; p = 0.047) and those who have had serious injury in the past 1 year (AOR: 2.29; 95% CI:1.78–2.95; p < 0.001) were significantly more likely to be involved in peer physical violence. Adolescents that were bullied had a 1.6 times higher odds of being involved in PPV than their peers (AOR: 1.70; 95% CI:1.28–2.25; p < 0.001) while those who were bully perpetrators had about twice higher odds of being involved in PPV than their counterparts (AOR: 1.89; 95% CI: 1.37–2.61; p < 0.001). Conversely, adolescents that were religious had a 0.7 times less odds of being involved in PPV (AOR: 0.70; 95% CI: 0.53–0.92; p = 0.011) (Table 4).

**TABLE 4:** Multivariable binary logistic regression of predictors of Peer Physical Violence among adolescents. N = 1,296 (Prevalence and Predictors of Peer Physical Violence among Adolescents in a Sub-national Region of Nigeria, Abakaliki, Nigeria; January 2021).

Variable	COR (95% CI)	AOR (95% CI)	p-value
Age group (Years)			
11–13	1	1	
14–16	0.63 (0.48–0.83)	0.63 (0.46–0.85)	0.002^a^
17–19	0.38 (0.27–0.52)	0.37 (0.25–0.53)	<0.001^a^
Alcohol intake (No)	1	1	
Yes	1.84 (1.44–2.36)	1.08 (0.80–1.46)	0.604
Drug/substance use (No)	1	1	
Yes	2.15 (1.61–2.88)	1.39 (0.98–1.97)	0.064
Cigarette smoking (No)	1	1	
Yes	4.09 (2.50–6.66)	1.85 (1.01–3.40)	0.047^a^
Passive smoking (No)	1	1	
Yes	1.61 (1.29–2.02)	1.14 (0.89–1.48)	0.291
Gambling (No)	1	1	
Yes	2.59 (1.96–3.43)	1.56 (1.13–2.16)	0.007^a^
Sexual exposure (No)	1	1	
Yes	1.60 (1.14–2.23)	1.09 (0.72–1.66)	0.674
Weapon carrying (No)	1	1	
Yes	3.00 (1.87–4.81)	1.08 (0.59–1.98)	0.806
Serious injury (No)	1	1	
Yes	2.86 (2.27–3.59)	2.29 (1.78–2.95)	<0.001^a^
Suicide attempt (No)	1	1	
Yes	2.69 (1.76–4.13)	1.44 (0.86–2.39)	0.162
Bully victim (No)	1	1	
Yes	2.95 (2.34–3.73)	1.70 (1.28–2.25)	<0.001^a^
Bully perpetrator (No)	1	1	
Yes	3.50 (2.70–4.53)	1.89 (1.37–2.61)	<0.001^a^
Religiosity (No)	1	1	
Yes	0.64 (0.50–0.81)	0.70 (0.53–0.92)	0.011^a^

^a^ Statistically significant; COR, crude odds ratio; AOR, adjusted odds ratio.

## Discussion

This study highlights the prevalence and predictors of peer physical violence, also known as physical fighting among school-attending adolescents in a sub-national region of Nigeria. The findings indicate that the prevalence of peer physical violence was 43.1%. In the multivariable model, being involved in gambling, being a bully-victim or bully-perpetrator, having had serious injury and cigarette smoking were associated with involvement in physical fighting. Furthermore, the two protective associations were the attributes of being an older adolescent and being religious.

The prevalence in this present study is less than the findings from an earlier study in Ghana [22], but comparable to another study from Egypt [9]. However, the prevalence of this study was more than that obtained in a previous study in Nigeria a decade ago [20]. This is possibly because the sample size for this present study is much more than that for the previous study. Another probable reason may be because PPV was not the primary variable assessed in the previous study; it was only one of the independent variables obtained. Therefore. It may not be that the prevalence of PPV has actually increased from the last decade.

Age was a strong predictor of peer physical violence in this study, and is consistent with findings in other studies around the world [17, 20, 23]. The higher prevalence of peer physical violence in younger adolescent ages may be attributed to the uncontrollable emotional feelings associated with onset of early adolescence as has been proposed by previous authors [17].

The finding that gambling was significantly associated with peer physical violence is in keeping with a finding from the United States [24] which related peer physical violence with more permissive gambling attitudes. This finding is important at present where there seems to be a proliferation of gambling sites and activities in Nigeria, with poor regulatory policies especially as it concerns adolescents [25].

Again, bullying was found to be a significant association with peer physical violence [8, 9]. Being a bully victim may make one more reactive to conflict by other people, and these victims may easily get into fights with their bullies, while attempting to defend themselves.

This study obtained a significant association between having a serious injury and peer physical violence, consistent with findings from several previous studies [13, 14, 26, 27]. It follows that persons who engage in peer physical violence will be at a greater risk of sustaining physical injuries.

Cigarette smoking was another factor that was significantly associated with adolescents who fight, as observed in this study. This correlates with previous studies in other parts of the world [28–30]. The clustering of adolescent health-risk behaviours as demonstrated by previous authors [18] suggests that adolescents who are likely to engage in physical fights are also likely to be engaged in other risky behaviours like cigarette smoking.

This study found an inverse relationship between involvement in peer physical violence and religiosity. This is in keeping with an earlier study [30] that explored the effect of religiosity on adolescent risk behaviours in England. This finding was also similar to a study carried out in the United States of America [31]. Being religious may offer more opportunity for adolescents to receive mentorship and guidance from adults in an organised setting like places of worship, as well as having some higher level of accountability compared to their peers who do not engage in religious activities.

### Limitation and Strength of the Study

The findings of this work would need to be interpreted knowing that this was a cross-sectional study in which it will not be possible to demonstrate the temporality of the relationship between the factors and physical fighting. However, the large sample size and the use of an internationally validated questionnaire are strengths of the study. In the future, it may be important to carry out a qualitative study as a way of providing further insights into potential social nuances around peer physical violence.

In conclusion, the prevalence of peer physical violence also known as physical fighting is high in Ebonyi State, Nigeria. The risk factors were young adolescent age, bullying, gambling, cigarette smoking and having had a serious injury. Being religious was significantly associated with protection. The findings of this study have provided evidence that can be used in designing and implementing interventions and policies aimed at curbing this pattern of violence.
