# Supplementary material for: Prevalence and Predictors of Peer Physical Violence Among Adolescents in a Sub-National Region of Nigeria
Source: Int J Public Health. 2025 Jul 17;70:1608128. doi: 10.3389/ijph.2025.1608128 (PMC12310562; doi:10.3389/ijph.2025.1608128)
Supplement: Supplementary file 1 [file Table1.docx]

### Appendix1: Independent variables definitions from the GSHS questionnaire

| Variable | Survey question | Coding |
| --- | --- | --- |
| Family socioeconomic status | The occupation of the father/male guardian and the highest level of education of the mother/female guardian of the study participants were collected. | Numbers 1 – 3 were assigned to the occupations, with 3 being for unskilled work and 1 for professionals. Highest level of education was assigned 0 – 2 with 0 for tertiary education and 2 for no formal and primary education. Finally, the sum of both was used to assign the social class as higher (1, 2), middle (3) or lower (4, 5). |
| Gambling | In the past 12 months, how many times were you involved in gambling? Defined to include but not limited to sports betting. | 1 or more = Yes (1)  None = No (0) |
| Cigarette smoking | During the past 30 days, on how many days did you smoke cigarettes? | 1 or more = Yes (1)  None = No (0) |
| Passive smoking | During the past 7 days, on how many days have people smoked in your presence? | 1 or more = Yes (1)  None = No (0) |
| Alcohol intake | During the past 30 days, on how many days did you have at least one drink containing alcohol?A ‘drink’ was defined as a glass of wine, a bottle of beer, a small glass of liquor or a mixed drink; and does not include taking a few sips of alcohol for religious purposes. | 1 or more = Yes (1)  None = No (0) |
| Weapon carrying | In the past 12 months, how many times did you carry a weapon e.g. penknife, gun, club, etc. to school for the purpose of attacking someone else or for defending yourself? | 1 or more = Yes (1)  None = No (0) |
| Serious injury | During the past 12 months, how many times were you seriously injured?Defined as when an injury makes you to miss at least one full day of activities or requires treatment by a doctor or a nurse. | 1 or more = Yes (1)  None = No (0) |
| Bullying victimization | During the past 30 days, on how many days were you bullied?Defined as when a student or group of students say or do bad and unpleasant things to another student. Also includes when a student is teased a lot in an unpleasant way or is left out of things on purpose. | 1 or more days = Yes (1)0 = No (0) |
| Bullying perpetration | During the past 30 days, on how many days did you bully someone? | 1 or more days = Yes (1)0 = No (0) |
| Drug use | During your life, how many times have you used drugs?Defined as the use of any substance or prescription drug for purposes other than those for which they are meant to be used, or in excessive amounts in order to ‘get high’. | 1 or more = Yes (1)Never = No (0) |
| Sexual exposure | Have you ever had sexual intercourse? | Yes = 1No = 0 |
| Suicide attempt | During the past 12 months, how many times did you actually attempt suicide? | 1 or more = Yes (1)Never = No (0) |
| Religiosity | Assessed using 3 questions. The first question assessed attendance to religious services and other religious activities, excluding attendance for wedding and burial events. Any attendance more than 2 in a month was assigned 2; 1 or 2 times = 1 and no attendance in the past one month = 0.  In review  The next two questions assessed perspective and attitude/belief. One question was “it is important to turn to prayer when you are facing a personal problem.” Answers ranged from strongly disagree to strongly agree.  The last question assessing religiosity was “it is important to rely on religious beliefs as a guide for your day to day living.” Answers for this also ranged from strongly disagree to strongly agree.  For these last two questions, strongly disagree and disagree were assigned 0, neutral = 1, agree and strongly agree  = 2. | An aggregate composite score was derived by adding the scores, with the minimum score being 0 and maximum score being 6. Any aggregate composite score above the mean score of 5.3 was taken as religious. |
